# Stigma and discrimination tendencies towards COVID-19 survivors: Evidence from a nationwide population-based survey in Ghana

**DOI:** 10.1371/journal.pgph.0000307

**Published:** 2022-06-22

**Authors:** Eric Osei, Hubert Amu, Prince Kubi Appiah, Solomon Boamah Amponsah, Evans Danso, Samuel Oppong, Comfort Worna Lotse, Bright Emmanuel Owusu, Simon Azure Agongo, Eliasu Yakubu, Gideon Kye-Duodu

**Affiliations:** 1 Department of Population and Behavioural Sciences, School of Public Health, University of Health and Allied Sciences, Hohoe, Ghana; 2 Department of Public Health, Yonsei University of Graduate School, Seoul, Republic of Korea; 3 Department of Family and Community Health, School of Public Health, University of Health and Allied Sciences, Hohoe, Ghana; 4 Department of Medical Law and Bioethics, Yonsei University, Seoul, Republic of Korea; 5 Department of Health Research and Development, Regional Health Directorate, Ghana Health Service, Eastern Region, Koforidua, Ghana; 6 Department of Community Health, College of Health, Yamfo, Bono East Region, Sunyani, Ghana; 7 Municipal Health Directorate, Ghana Health Service, Bono Region, Sunyani, Ghana; 8 Department of Nursing, School of Nurses and Midwifery, University of Health and Allied Sciences, Ho, Ghana; 9 Department of Mathematics, Kwame Nkrumah University of Science and Technology, Kumasi, Ghana; 10 Department of Epidemiology and Biostatistics, University of Health and Allied Sciences, Ho, Ghana; University of the Witwatersrand, SOUTH AFRICA

## Abstract

Historically, infectious diseases have generated fears among populations. Unhealthy handling of these fears result in the stigma and discrimination of infected patients. Globally, measures taken so far by governments to curb the spread of the novel coronavirus disease-2019 (COVID-19) pandemic, although helpful, have created fears in people. Consequently, there are reported Ghanaian media cases of stigmatisation against persons who were infected and recovered from COVID-19. However, these reports remain unsubstantiated. This study, therefore, sought to examine stigma and discriminatory tendencies towards COVID-19 survivors among the adult population in Ghana. This was a population-based cross-sectional study among 3,259 adults. A multi-stage sampling technique was used to recruit study participants. Descriptive and inferential statistics comprising frequency, percentage, chi-square, and multivariable logistic regression were employed in analysing the data. Knowledge on COVID-19 was poor among 33.6% of the participants. Forty-three per cent had a good attitude towards COVID-19. Nearly half (45.9%) exhibited stigma and discriminatory tendencies towards COVID-19 survivors. Participants who had poor COVID-19 related knowledge (aOR = 1.91, 95%CI = 1.59–2.29, p<0.001) and poor attitude towards COVID-19 (aOR = 5.83, 95% CI = 4.85–6.98, *p*<0.001) were more likely to exhibit stigma and discriminatory tendencies towards COVID-19 survivors. Our study found relatively high proportions of poor knowledge and negative attitudes towards COVID-19. Stigma and discriminatory tendencies were consequently high. Our findings call for increased public education on COVID-19 by the Ghana Health Service and the Information Services Department, to increase the level of knowledge on the pandemic while reducing stigma and discrimination associated with it.

## Background

The storm-like outbreak of the novel coronavirus disease-2019 (COVID-19), which began in Wuhan, China in late 2019, has had a huge impact on human health; infecting millions of people; causing severe disease and associated long-term sequelae; resulting in several deaths and excess mortality worldwide [1]. The outbreak has since created fear and panic among the populace, which has led to an increase in acts of stigma, discrimination, and xenophobia towards people associated with the disease [2, 3].

Stigma refers to “the negative regard, inferior status, and relative powerlessness that society collectively accords to people who possess a particular characteristic or belong to a particular group or category” [4]. There are six dimensions of stigma which entail origin, course, concealability, disruptiveness, aesthetics, and peril [5]. Stigma is not new to public health. History provides several instances of prejudice and discrimination against people with specific diseases [6–8], which has been associated with a lack of knowledge about how a disease spreads, a need to blame someone, fears about disease transmission and death, and gossip that spreads rumours and myths. Tuberculosis, Human Immuno-Deficiency Virus (HIV)/ Acquired Immune Deficiency Syndrome (AIDS), and leprosy for instance are well-known stigmatised infectious diseases. More recently, survivors of the Ebola outbreak in the Westen part of Africa faced discrimination and unemployment when they returned to their communities [9].

Stigmatisation has the potential to negatively affect preventive, diagnostic and therapeutic efforts to combat diseases. Evidence has shown that stigma due to COVID-19 leads to a reduction in people seeking medical care or testing and a reduction in people adhering to interventions, which may lead to underreporting of cases [10]. In the Gambia for instance, a growing number of people were reluctant to visit health facilities, dreading the implications of being pigeonholed as carriers of coronavirus [11].

The level of stigma associated with COVID-19 may be as a result of three main factors; 1) It is a disease that is new and for which there are still many unknowns; 2) Being afraid of the unknown and; 3) Easy to associate the fears with others. Stigma can drive people to hide the illness to avoid discrimination, prevent people from seeking prompt health care and discourage them from adopting healthy behaviours [11]. When not checked, stigma can undermine social cohesion and prompt possible social isolation of groups which can contribute to more serious health problems, worsening the ongoing transmission, making it much harder for health authorities to control the outbreak [10, 11].

In Ghana, matters of stigma and discrimination against people living with certain diseases including HIV/AIDS, Tuberculosis (TB), Leprosy, mental illnesses are not uncommon [12]. Asampong et al. [12] argued that stigma and discriminatory tendencies for diseases in Ghanaian societies are mainly influenced by cultural and social interpretations put on these diseases. According to them, the tendency to name diseases that provoke apprehension, fear or negative connotations within the Ghanaian society tends to enrich the stigma and discrimination against persons living with such diseases.

There have been several media reports of stigma and discrimination towards COVID-19 patients and their family members since the first case was reported in Ghana in March 2020. A survivor of the disease for instance bemoaned on a radio station in Ghana regarding what he considered stigmatisation towards him and the family. Recounting his experience during a press briefing organised by the Government of Ghana through the Ministry of Information, he said people refused to have contact with him and his wife even after recovery. Their children were not left out, as the other children in the neighbourhood pointed fingers at them when they passed by [13]. Adogla-Bessa [14] also claimed that a recovered patient and his family were being stigmatised by his community members to the extent of closing their shops on them or refusing to sell items to them. These worrying stories compelled the President to call on Ghanaians to stop stigmatisation and discrimination towards victims of the disease, indicating that COVID-19 is not a death sentence [15].

Empirical studies that have been conducted in Ghana on COVID-19 related stigma include those by Adom et al. [16], Atinga et al. [17], Lamptey et al. [18] and Bandoh et al. [19]. The authors argued that COVID-19 stigma is mainly meted out to survivors and their contacts, including being avoided, having derogatory words used on them, or being discriminated against by their friends and community members. Ghanaian returnees from high hurden COVID-19 countries especially those from Asia (including China) have also experienced stigma. These studies, however, focused on small sections of the population. Lamptey et al. [18], for instance, assessed stigmatising attitudes towards COVID-19 survivors among 290 non-representative samples of Ghanaians and 220 Nigerians. Atinga et al. assessed the experiences of 45 COVID-19 Survivors and their Coping Pathways related to stigma, discrimination, social exclusion. The current study, therefore, sought to examine the predictors of COVID-19 related stigma and discrimination attitudes among a representative Ghanaian population. The study will be essential in adding to the existing literature on the magnitude and predictors of COVID-19 related stigma in the country.

## Methods

### Study design and population

A population-based descriptive cross-sectional design was employed for the conduct of this study. The study population included Ghanaian adults aged 18 years and above, who responded to the survey questionnaire.

### Study site

Ghana is a country in western Africa just north of the Equator. The country is situated between Cote d’Ivoire and Togo and bordered by Burkina Faso in the north and by the Gulf of Guinea (Atlantic Ocean) in the south. The most densely populated parts of the country are the coastal areas and Kumasi, the Ashanti regional capital. Ghana’s population of about 30 million spans a variety of ethnic, linguistic, and religious groups residing in 216 districts distributed among 16 administrative regions [20].

Ghana reported its first two cases of COVID-19 on March 12, 2020. This rose to 141 cases in just two weeks, affecting three regions. The disease subsequently spread to all 16 regions of the country a few weeks later, with the Greater Accra and Ashanti regions, the most populous regions in the country, being the "hot spots". As of 27th August 2021, the country had reported 116,441 confirmed cases to the WHO, representing about 2% of the total cases reported from the WHO’s African region and ranked 11th in the region in terms of case count. Of these, 991 died, representing a CFR of 0.85% [21].

### Sample size determination

Cochran’s formula for determining sample size was used to estimate the final sample size for each selected region [22]. The formulae is given as:

$$n=\frac{z^{2}p(1-p)}{e^{2}}$$

where *n* is the minimum sample size, *z* is the value for the selected alpha level of 1.96 (0.25 in each tail) at 95% confidence level, *p* is the estimated proportion of an attribute that is present in the population (0.5), and *e* is the acceptable margin of error for proportion being estimated (0.05).

Based on this formulae, the minimum sample size required for each region was 384 and with a non-response rate of 5%, 404 participants were selected from each region. The total sample size required in this study was 3,636.

### Sampling procedures

The study utilised a sampling frame as an updated frame from the 2010 Ghana Population and Housing Census (PHC) that was obtained from the Ghana Statistical Service. The sampling frame excluded nomadic and institutional populations such as persons in hotels, barracks, and prisons. A multi-stage sampling design intended to allow estimation of key variables at the national level as well as for urban and rural areas and each of the sampled administrative regions of Ghana was adopted. At the first stage, the 16 administrative regions of Ghana were stratified into three ecological zones namely, 1) Savanna Zone (consisting of Upper West, Upper East, Northern, North-east, and Savanna regions; 2) Middle Zone (comprising of Bono, Ahafo, Bono East, Ashanti, and Eastern regions); and 3) Coastal Zone (Greater Accra, Central, Western, Western North, Volta, and Oti regions). For zonal comparisons, a lottery type of simple random sampling method was used to select 3 regions from each zone. This was achieved by writing the names of the regions in each zone on pieces of paper which were then folded and put into separate containers. The containers were shaken, and three papers were picked after each shaking of the boxes.

The second stage involved a simple random sampling of 2 districts (1 rural and 1 urban) from each of the selected regions using the same process above. In the third stage, a random sample of 30 clusters from each participating district was selected through a lottery method. The sample size for each region was proportionally divided among the 30 clusters according to population size. The final stage was the selection of housing units where the random walk technique was adopted. In each housing unit, one eligible respondent was selected randomly through the lottery method to take part in the survey.

### Data collection instruments and measures

Pretested questionnaires were used to capture data. The questionnaires, which captured sociodemographic (age, sex, marital status, occupation, education, religion, income level and, residence) information of respondents; social stigma, and discriminatory attitudes that measured the fear of casual transmission and refusal of contact; knowledge of disease transmission and signs and symptoms; and general attitude towards COVID-19 were pretested in a community in each region (not included in the study) before the actual data collection. S1 Table is the questionnaire used.

Five items that were adopted from the USAID’s recommended indicators and questions for measuring HIV/AIDS-related stigma and discrimination [23] were used to measure stigma and discrimination tendencies. Response categories for stigma items included strongly disagree, disagree, neutral, agree and strongly agree (5-point Likert scale). To guide against acquiescence and carelessness biases, a mixture of positive and negative-worded stigma items were used. Internal consistency and the reliability of the stigma items were measured using Cronbach’s alpha coefficient and an alpha equal to or greater than 0.70 was considered satisfactory. COVID-19 related knowledge and general attitude were measured using 9 items each.

Experienced social science researchers, before data collection, reviewed the instrument for syntax, organisation, and appropriateness and confirmed that it appeared to flow logically and validated that the items were appropriate indicators of each construct measured.

### Data collection procedure

Data collection for this study was carried out from October to November 2020. Data collection was done in the form of computer-assisted personal interviews (CAPI). These were in the form of questionnaires installed on the smartphones of research assistants. The data were collected by health personnel with at least a tertiary educational background and understood and can speak the most common local language of the district. This personnel received two-day intensive training on interview techniques and how to administer the study tools before the data collection. During the data collection, the data collectors visited households as early as about 6 am each day to meet the working force and farmers at home before they left for work. The principal investigator along with co-investigators strictly supervised the overall data collection procedure during fieldwork.

### Data handling and analysis

Data were extracted from the online database in an excel format for cleaning. Descriptive statistics such as mean or median, frequencies, and proportions were used to describe and summarise the quantitative data. Stigma items that were reverse-worded were reverse-scored before data were analysed.

COVID-19 related knowledge and attitude were measured with ‘yes’, ‘no’ and ‘do not know’ responses and a combination of dichotomous (yes, no) and Likert (strongly agree, agree, neutral, disagree, strongly disagree) rating scales, respectively, and were scored as ‘1’ (correct) and ‘0’ (wrong), with scores ranging from 0–8 each. Regarding knowledge, ‘Do not know’ responses were considered as lack of knowledge and were scored as ’0’. The 5-Likert attitude items were dichotomised by combining ‘strongly agree’ and ‘agree’ = 1 and ‘strongly disagree’ and ‘disagree’ = 0. Knowledge scores above the mean value were considered good knowledge, while scores below the mean were considered poor knowledge, likewise attitude.

Five answerable questions were indexed to assess the level of stigma and discrimination intensions of the respondents. Respondents were assigned a score of “1” for a positive stigma attitude and a score of “0” for a negative stigma attitude. Cumulated scores were then converted to percentages. Individuals who scored above the average stigma score were considered to have “No stigma” and those who had lower than average percentage scores were classified to have stigma and discriminatory tendencies.

Multiple logistic regression analysis was done to identify significant predictors of stigma and discrimination after controlling for other explanatory variables. The variables found to be significantly (p<0.20) associated with the outcome variable in the bivariate models were entered into a final model. All analyses were done using Stata Version 13.1. S1 Data contains the study data.

### Ethical considerations

Ethical approval was obtained from the Research Ethics Committee (REC) of the University of Health and Allied Sciences (UHAS) (Reference No. UHAS-REC A.9 [4] L9-20). Written informed consent was obtained from all participants.

## Results

### Socio-demographic characteristics

Table 1 presents the sociodemographics of the study participants. Out of the 3636 participants approached for the survey from 18 districts of Ghana, 3,529 participated, representing a 97.1% response rate. Of these, more than half (57.3%) resided in urban areas with the majority (43.1%) between 20 and 29 years of age with a median age of 29 years. Besides, male participants constituted 54.9% and 44.1% had secondary school education while students were 28.5% of the respondents.

**Table 1:** Socio-demographic characteristics of respondents.

Variable	Rural	Urban	Total
	(N = 1508)	(N = 2021)	(N = 3529)
	n (%)	n (%)	n (%)
Age group (years)			
18–20	126 (43.5)	164 (56.5)	290 (8.2)
20–29	596 (39.2)	925 (60.8)	1521 (43.1)
30–39	462 (48.22)	496 (51.8)	958 (27.1)
40–49	198 (43.5)	257 (56.5)	455 (12.9)
50–59	89 (41.4)	126 (58.9)	215 (6.1)
60+	37 (41.1)	53 (58.6)	90 (2.6)
Sex			
Male	832 (42.9)	1106 (57.1)	1938 (54.9)
Female	676 (42.5)	915 (57.5)	1591 (45.1)
Education*			
Tertiary	549 (38.4)	879 (61.6)	1428 (40.5)
JHS/SHS	673 (43.2)	884 (56.8)	1557 (44.1)
Primary	117 (57.4)	87 (42.6)	204 (5.8)
None	169 (49.7)	171 (50.3)	340 (9.6)
Occupation			
Government worker	411 (43.3)	538 (56.7)	949 (26.9)
Work for someone/company	138 (35.4)	252(64.6)	390 (11.0)
Self-employed	365 (49.3)	375(50.7)	740 (21.0)
Student	391 (38.9)	614 (61.1)	1005 (28.5)
Unemployed	203 (45.6)	242 (54.4)	445 (12.6)
Marital Status			
Married	631 (43.9)	805 (56.1)	1436 (40.7)
Widowed/widower	58 (43.0)	77 (57.0)	135 (3.8)
Co-habiting	99 (47.6)	109 (52.3)	208 (5.9)
Single	720 (41.1)	1030 (58.9)	1750 (49.6)
Religion			
Christian	974 (41.4)	1375 (58.6)	2349 (66.6)
Moslem	44 0(44.4)	550 (55.6)	990 (28.1)
Traditionalist	63 (47.7)	69 (52.3)	132 (3.7)
Other	31 (53.4)	27 (46.6)	58 (1.6)
Monthly Income(GH¢)			
<355	727 (46.7)	830 (53.3)	1557 (44.1)
355–500	142 (41.3)	202 (58.7)	344 (9.7)
501–1000	214 (38.2)	346 (61.8)	560 (15.9)
1001–2000	307 (43.1)	405 (56.9)	712 (20.2)
2000+	119 (37.4)	199 (62.6)	318 (9.0)
Not specified	0 (0.0)	38 (100.0)	38 (1.1)
Region			
Ashanti	102(26.5)	283 (73.5)	385 (10.9)
Bono East	145(37.7)	240 (62.3)	385 (10.9)
Eastern	229(57.3)	171 (42.7)	400 (11.3)
Greater Accra	101(26.2)	284 (73.8)	385 (10.9)
Northern	229(56.5)	176 (43.5)	405 (11.5)
Upper East	53(13.2)	348 (86.8)	401 (11.4)
Upper West	141(35.4)	257 (64.6)	398 (11.3)
Volta	265(68.8)	120 (31.2)	385 (10.9)
Western North	243(63.1)	142 (36.9)	385 (10.9)

*represent the highest level of education completed or currently attending; SHS/JHS: Senior High School/Junior High School.

### Knowledge on COVID-19

Table 2 presents the results of COVID-19 knowledge by item. The item with the highest rate (83.9%) of correct answers was “Coronavirus can be transmitted through the aerosol of infected person” and the item with the lowest rate (65.4%) of correct responses was "There is a specific treatment for COVID-19”. A considerable proportion (11.9%) of the survey respondents wrongly answered that mosquitoes can transmit COVID-19. The mean knowledge score was 4.9±1.69. Overall, 66.4% (2,343) of respondents had good knowledge regarding COVID-19, while the rest had poor knowledge.

**Table 2:** COVID-19 related knowledge among respondents.

Knowledge item	Yes	No	Don’t know
	n (%)	n (%)	n (%)
Coronavirus can be transmitted through the air	2508 (71.1)	821 (23.3)	200 (5.6)
Coronavirus can be transmitted through the aerosol of an infected person	2964 (84.0)	228 (6.5)	337 (9.5)
Mosquitoes can transmit the coronavirus	420 (11.9)	2530 (71.7)	579 (16.4)
Coronavirus can be transmitted by an asymptomatic person	2919 (82.7)	266 (7.5)	344 (9.8)
A person who recovers from COVID-19 can be re-infected	2464 (69.8)	500 (14.2)	565 (16.0)
People who have been infected with the coronavirus may be asymptomatic	2721 (77.1)	463 (13.1)	345 (9.8)
COVID-19 has specific treatment	2308 (65.4)	763 (21.6)	458 (13.0)
Coronavirus is a curse from God	607 (17.2)	2145 (60.8)	777 (22.0)

### Attitude towards COVID-19

Table 3 presents attitude towards COVID-19. Of the 3,529 participants, 9.4% had been tested for COVID-19. Of those who had not been tested, 73.3% expressed willingness to take the test if there is an opportunity to do so whereas 2% were not sure whether they want to be tested or not. Of all survey respondents, 45.5% would not disclose their positive COVID-19 test results to friends and/or co-workers; about half (49.7%) of them would be surprised if they were tested positive for COVID-19, and nearly one-third (30.6%) feared to visit any health facility due to the fear of COVID-19 transmission. The mean attitude score was 4. 7 ± 2.04. Generally, 57% (2013) of the respondents had a good attitude towards COVID-19 and the rest had a poor attitude.

**Table 3:** COVID-19 related attitudes of respondents.

Attitude item	n	%
Have taken the COVID-19 test		
Yes	333	9.4
No	3030	85.9
Prefer not to say	166	4.7
Willingness to take COVID-19 test (N = 3030)		
Yes	2192	72.3
No	777	25.7
Undecided	61	2.0
Willingness to disclose COVID-19 positive test result		
Yes	1922	54.5
No	1607	45.5
I Will be surprised if tested positive for COVID-19		
Yes	1775	50.3
No	1754	49.7
At risk of COVID-19		
Yes	2086	59.1
No	1443	40.9
Fear of visiting a health facility if ill due to COVID-19		
Yes	1079	30.6
No	2450	69.4
I am willing to run errands for COVID-19 patients in isolation		
Strongly agree	511	14.5
Agree	1416	40.1
Neutral	521	14.8
Disagree	663	18.8
Strongly disagree	418	11.8
Names of all people who tested positive for Coronavirus should be made public		
Strongly agree	328	9.3
Agree	541	15.3
Neutral	508	14.4
Disagree	1126	31.9
Strongly disagree	1026	29.1
I would not mind if the COVID-19 Isolation centre is near my vicinity		
Strongly agree	589	16.7
Agree	1521	43.1
Neutral	558	15.8
Disagree	464	13.1
Strongly disagree	397	11.3
I will attend to a COVID-19 patient if I were a Nurse or Doctor		
Strongly agree	1174	33.8
Agree	1688	47.8
Neutral	326	9.2
Disagree	208	5.9
Strongly disagree	133	3.8

### Prevalence of stigma and discrimination

Table 4 presents the prevalence of stigma and discriminatory tendencies among the participants. Five-point Likert’s items were used to assess stigma and discriminatory tendencies related to COVID-19 survivors. The internal consistency reliability of the instrument was assessed using Cronbach’s alpha and has a reliability coefficient of 0.82. More than one-tenth (12.5%) of respondents would not agree to stay in the same house with someone who recovers from COVID-19; 21.8% would not work together with them and 20.6% said they would dismiss their employees who recover from COVID-19 if they were employers while 13.5% referred to be neutral about that. Overall, 45.9% of participants exhibited stigma and discriminatory tendencies towards COVID-19 survivors.

**Table 4:** Stigma and discriminatory tendencies towards COVID-19 survivors.

Stigma and discrimination items	Strongly agree	Agree	Neutral	Disagree	Strongly disagree
	n (%)	n (%)	n (%)	n (%)	n (%)
I will stay in the same house with a COVID-19 survivor	613(17.4)	1896(53.7)	579(16.4)	274(7.8)	167(4.7)
I will be willing to sit in the same vehicle with a COVID-19 survivor	580(16.4)	1916(54.3)	559(15.8)	324(9.2)	150(4.3)
I will avoid working together with a COVID-19 survival	171(4.9)	598(16.9)	552(15.6)	1506(42.7)	702(19.9)
I will avoid eating together with a COVID-19 survivor	235(6.6)	718(20.4)	618(17.5)	1366(38.7)	592(16.8)
I will dismiss my employee who recovers from COVID-19	484(13.7)	242(6.9)	478(13.5)	1518(43.0)	807(22.9)

### Predictors of stigma and discrimination towards COVID-19 survivors

Table 5 presents results of binary logistic regression analysis of factors associated with stigma and discrimination intention towards COVID-19 survivors. After adjusting for the confounding effect of each predictor variable, respondents who were between 30 and 59 years old were more likely to have stigma and discrimination intention compared to those who were 18–29 years old. Further, respondents with between 501 and 2000 cedis as their monthly income were more likely to stigmatise COVID-19 survivors compared to their counterparts with a monthly income of fewer than 355 cedis. Those who lived in Bono East and Upper West regions had lower odds of having stigma and discrimination intention whereas participants who resided in the Northern region had 75% higher odds compared to those who lived in the Ashanti region. Respondents who had poor COVID-19 related knowledge (aOR = 1.91, 95% CI = 1.59–2.29, *p* < 0.001) and poor attitude towards COVID-19 (aOR = 5.83, 4.85–6.98, p<0.001) were more likely to exhibit stigma and discriminatory tendencies towards COVID-19 survivors.

**Table 5:** Predictors of stigma and discriminatory tendencies towards COVID-19 survivors.

Variable	Stigma and discriminatory tendency	cOR (95%CI)	aOR* (95%CI)	P-value
	n(%)			
Age group (years)				
18–29	821 (45.3)	ref	ref	
30–39	419 (43.7)	0.94 (0.80–1.09)	1.34 (1.06–1.70)	0.013
40–49	227 (50.0)	1.21 (0.98–1.48)	1.59 (1.17–2.17)	0.003
50–59	110 (51.2)	1.26 (0.95–1.68	1.54 (1.02–2.31)	0.036
60+	43 (47.8)	1.10 (0.72–1.69)	0.99 (0.57–1.74)	0.990
Sex				
Male	8843 (43.5)	ref	ref	
Female	777 (48.8))	1.23 (1.08–1.41)	1.10 (0.94–1.29)	0.230
Education				
Tertiary	548 (38.4)	ref	ref	
Secondary	760 (48.8)	1.53 (1.67–3.02)	1.19 (0.92–1.54)	0.181
Primary	119 (58.3)	2.25 (1.67–3.02)	1.34 (0.89–2.02)	0.160
No formal education	193 (56.8)	2.11 (1.65–2.68)	1.14 (0.79–1.64)	0.496
Occupation				
Unemployed	705 (48.6)	ref	ref	
Government worker	346 (36.5)	0.61(0.51–0.72)	0.88 (0.63–1.25)	0.498
Work for someone	176 (45.1)	0.87 (0.69–1.08)	0.93 (0.68–1.25)	0.620
Self-employed	393 (53.1)	1.19 (1.00–1.43)	0.91 (0.70–1.19)	0.509
Marital Status				
Currently married	638 (44.5)	ref	ref	
Never married	900 (45.9)	1.06 (0.93–1.22)	1.20 (0.96–1.52)	0.115
Ever married	82 (60.7)	1.93 (1.34–2.78)	1.40 (0.89–2.21)	0.149
Religion				
Christian	1024 (43.6)	ref	ref	
Moslem	486 (49.1)	1.25 (1.08–1.45)	0.96 (0.79–1.17)	0.699
Traditionalist	85 (64.9)	2.34 (1.62–3.37)	1.19 (0.75–1.87)	0.458
Other	25 (43.9)	1.01 (0.59–1.27)	0.59 (0.32–1.10)	0.100
Monthly Income				
<355	760 (48.8)	ref	ref	
355–500	159 (46.2)	0.90 (0.71–1.13)	1.02 (0.77–1.36)	0.887
501–1000	252 (45.0)	0.86 (0.71–1.04)	1.35 (1.02–1.79)	0.036
1001–2000	283 (39.8)	0.69 (0.58–0.82)	1.37 (1.01–1.85)	0.041
2000+	146 (45.9)	0.89 (0.69–1.13)	1.37 (0.95–1.98)	0.092
Not specified^+^	19 (50.0)	-	-	-
Region of residence				
Ashanti	191 (49.6)	ref	ref	
Bono East	100 (26.0)	0.35 (0.26–0.48)	0.42 (0.29–0.59)	<0.001
Eastern	242 (60.5)	1.56 (1.17–2.06)	1.24 (0.89–1.72)	0.194
Greater Accra	169 (43.9)	0.79 (0.59–1.06)	0.94 (0.68–1.29)	0.695
Northern	256 (63.2)	1.75 (1.31–2.32)	1.73 (1.24–2.43)	0.001
Upper East	252 (62.8)	1.72 (1.29–2.28)	1.34 (0.96–1.86)	0.080
Upper West	54 (13.6)	0.16 (0.11–0.23)	0.27 (0.19–0.41)	<0.001
Volta	171 (44.4)	0.81 (0.61–1.07)	0.91 (0.65–1.26)	0.566
Western North	185 (48.1)	0.94 (0.71–1.24)	0.91 (0.66–1.27)	0.592
Residential status				
Urban	906 (44.8)	ref	ref	
Rural	714 (47.4)	1.11 (0.96–1.27)	1.00 (0.84–1.19)	0.976
COVID-19 Risk perception				
At-risk	884 (42.4)	ref	ref	
Not at risk	585 (53.6)	1.57 (1.35–1.82)	0.65 (0.54–0.79)	<0.001
Don’t know^+^	151 (43.1)	-	-	-
COVID-19 related knowledge				
Good Knowledge	872 (37.2)	ref	ref	
Poor Knowledge	748 (63.0)	2.88 (2.49–3.33	1.91 (1.59–2.29)	<0.001
COVID-19 related attitude				
Good Attitude	540 (26.8)	ref	ref	
Poor Attitude	1080 (71.2)	6.75 (5.82–7.83)	5.83 (4.85–6.98)	<0.001

cOR: Crude Odds Ratio; aOR: Adjusted Odds Ration; + removed from the model due to collinearity; adjusted for all predictor variables.

## Discussion

Together with the recent ongoing COVID-19 pandemic, there is a growing negative social perception towards COVID-19 patients and their families. The degree of such perception might be different in different locations, but its presence in society is undeniable [24]. This current study examined stigma and discriminatory tendencies towards COVID-19 survivors among the adult Ghanaian population. Surveying stigmatising attitudes and behaviours in the community has far-reaching implications for improving such attitudes and behaviours in society. Findings from this study are, thus, instrumental in the implementation of behaviour change communication strategies.

The results showed that close to half of the respondents exhibited stigma and discriminatory tendencies towards COVID-19 survivors, which confirm the many personal experiences by some of these victims and media reports in the country and across the world about the existence of stigma and discrimination in this pandemic situation [17, 18, 25]. Lamptey et al. [18] for instance reported that notwithstanding the availability of scientific information and real-time news on the illness, stigma remains a major challenge in society. This is a worrying situation and calls for urgent and pragmatic measures to deal with it as its negative consequences on the response efforts and individual health cannot be underestimated. For instance, it is noteworthy that persons with COVID-19 may develop poor health-seeking behaviours such as avoiding testing because, by anticipating that they will be stigmatised by society, even when they have recovered from the disease, they may perceive the risk of losing their jobs and face social marginalisation [26]. This reactive behaviour facilitates the spreading of the virus, particularly among those with mild symptoms who fail to seek medical attention in order not to raise suspicion of their status [24]. Apart from easing transmission, stigma and discrimination may also have psychological consequences on the victims [16].

These stigmatising behaviours may be driven by fear, inadequate information regarding the appropriate scientific facts, and rumours at least in the initial months of the pandemic. Public education has appeared to be inadequate; the general public may not still have access to correct, reliable, and scientific sources of information or these are lost amid many confusing messages in the news and social media [25, 27, 28]. The spread and maintenance of stigma may partly be due to the frequent change of information about the transmission, treatment modalities, and outcomes of the coronavirus [28].

Our findings where the people with poorer COVID-19 knowledge and attitude were more likely to stigmatise and discriminate against COVID-19 survivors confirm previous findings by Roozenbeek et al. [29] and Plan International [30] in this regard. The reason could be that these groups of people are less informed or misinformed about COVID-19 and hence known little about the transmission of the disease from survivors to non-infected persons or have misconceptions and fear of people who have recovered from COVID-19, as they hold the belief that the survivors could still pose a health risk to others. This observation calls for more efforts from the government and partners to increase education among the general population because continuous discrimination or stigmatisation against the survivors can prevent persons who might have been infected by the virus from declaring their status or seeking care [31, 32].

Stigma and discriminatory tendencies increased with age in this study. These observations could be partly due to the fear of transmission of the virus and death by the older adults as the items used in assessing stigmatisation in this study are related to fear of transmission. Scholars have established that the risk of COVID-19 infection, hospitalisation, and death has been associated with increased age [33–38]. Those 60 years and above exhibited decreased stigma and discriminatory tendencies towards COVID-19 survivors though no statistically significant. This could be due to the reason that the elderly are more likely to have compassion and sympathy towards the sick and not stigmatise nor discriminate against people who recover from their illness.

Our study further showed that persons who showed optimism about their COVID-19 risk were less likely to stigmatise COVID-19 survivors. Persons who have a lower perception of the COVID -19 threat tend to believe that the condition is only reserved for categories of people such as the rich or whites [39–42]. Others also believe that COVID-19 is just a myth that does not exist in reality or is rhetorically fabricated by politicians to make money [43, 44]. This chunk of misconception could be attributed to a lack of effective risk communication, the rapid propagation of misinformation and misconception on the social media space and among the general population regarding COVID-19 [45]. As a result, they tend to discriminate or stigmatise people affected by the COVID-19 or survivors of the disease [32, 46]. The lower perception of risk and discrimination toward COVID-19 survivors could have grave implications in the rapid spread of the disease among the general population, because people who do not believe to be at risk of contracting COVID-19 will be probably less likely to adhere to all the preventive protocols including wearing of masks, physical distancing or washing of hands put in place by the health authorities to curb the spread of the pandemic while the infected will be reluctant in seeking health care or declaring their status, thus increasing the chance of the transmission of the disease to others as argued in previous studies by Roozenbeek et al. [29] and Stanley et al. [47].

Regarding the area of residence, people who resided in the Upper West and Bono East regions were less likely to exhibit stigma and discriminatory tendencies towards COVID-19 survivors compared to those who resided in the Ashanti region. The reason for the regional differences in the stigma and discriminatory tendencies could be attributed to the intensity of disease transmission. As at the time of this study, the Ashanti region was the second region with the highest COVID-19 case count behind the Greater Accra Region. Therefore, residents in the region are likely to stigmatise people associated with the disease due to fear of transmission. Nothern regional residents had 73% increased odds of exhibiting stigma and discriminatory tendencies towards COVID-19 survivors than those in the Ashanti region even though transmission of the disease was comparatively far lower in the Nothern region. The reason for this difference cannot be directly inferred and requires further investigation. Socio-demographic characteristics of respondents such as education, marital status, employment, and religion did not significantly predict stigma and discriminatory tendencies towards COVID-19 survivors.

### Strengths and limitations

The strength of this study lies in its large representative sample, resulting in strong external validity in relation to the target population. As such, the findings from the study can be generalised to the Ghanaian populace. Despite the important findings made in the current study and its strengths, its limitations are worth noting. First of all, as a cross-sectional study, we were unable to measure the causal relationship between variables. Secondly, the knowledge, attitude and stigma scales were not scientifically validated. All the same, experienced social scientists reviewed the instrument and validated that the items were appropriate indicators of each construct measured. Thirdly, we measured only the externalisation of the stigma that is associated with fear of transmission and refusal of contact, which represent only one dimension of stigma.

## Conclusion

Stigma and discriminatory tendencies were considerably high and associated with risk perception, poor COVID-19 related knowledge and attitude, and area of residence. Our findings thus call for well-planned social education to deal with stigma. Focused attention and preparedness to emerging stigma and discrimination and prompt response may thwart their spread and influence on the public mindset and behaviour. Myth busters from reliable sources may contribute to addressing stigma. In this age of infodemics, information about the appropriate and reliable sources is important. By addressing stigma in the populace through repetitive information on the illness and its effects in local languages through multiple channels, there is an increased possibility of effectiveness in the improvement of knowledge and attitudes towards the diseases. This will eventually result in reduced stigma and discrimination in the population. Future studies could consider measuring other dimensions of stigma e.g, internalised stigma. Further studies could also add qualitative interviews to obtain a more detailed understanding of the COVID-19 stigma and discrimination situation in Ghana.

## Supporting information

S1 Table Questionnaire. (DOCX)

S1 Data Study data. (DTA)
