# Supplementary material for: Stigma and discrimination tendencies towards COVID-19 survivors: Evidence from a nationwide population-based survey in Ghana
Source: PLOS Glob Public Health. 2022 Jun 22;2(6):e0000307. doi: 10.1371/journal.pgph.0000307 (PMC10021222; doi:10.1371/journal.pgph.0000307)
Supplement: S1 Table — (DOCX) [file pgph.0000307.s001.docx]

**Social stigma, discrimination, and Xenophobic attitudes among associated with COVID-19 IN Ghana: implications for outbreak response**

| **Sr.No.** | **Question** | **Responses** | **skip** |
| --- | --- | --- | --- |
|  | **Background information** |  |  |
| 001 | What is your age in years? | ………………………… |  |
| 002 | What is your sex? | 1. Male 2. female |  |
| 003 | What is your highest level of education? | 1. No formal education 2. Primary 3. High school 4. Tertiary |  |
| 004 | What is your occupation? | 1. Not employed 2. Government worker 3. Working for someone 4. Self-employed |  |
| 005 | What is your marital status? | 1. Single 2. Married 3. Widowed/widower 4. Co-habiting |  |
| 006 | what is your religion? | 1. Christian 2. Moslem 3. Traditionalist |  |
| 007 | What is your monthly income? | ………………………………. |  |
| 008 | what region are you currently living in? | ………………………………….. |  |
| 009 | How will you describe where you are currently living? | 1. Rural 2. Urbal |  |
| **Knowledge on COVID-19** | | | |
| 010 | Coronavirus can be transmitted through the air | 1. Yes 2. No 3. Don’t know |  |
| 011 | Coronavirus can be transmitted through aerosol of infected person | 1. Yes 2. No 3. Don’t know |  |
| 012 | A mosquito can transmit the coronavirus | 1. Yes 2. No 3. Don’t know |  |
| 013 | Coronavirus can be transmitted by an infected person who is not showing signs and symptoms | 1. Yes 2. No 3. Don’t know |  |
| 014 | People who have been infected with the coronavirus may not show signs and symptoms | 1. Yes 2. No 3. Don’t know |  |
| 015 | A person who recovers from the coronavirus can be infected with the virus again | 1. Yes 2. No 3. Don’t know |  |
| 016 | A person who recovers from the coronavirus can infect others | 1. Yes 2. No 3. Don’t know |  |
| 017 | There is treatment for Coronavirus | 1. Yes 2. No 3. Don’t know |  |
| 018 | The coronavirus pandemic is a curse from God | 1. Yes 2. No 3. Don’t know |  |
| 019 | what are the signs/symptoms of COVID-19? (check all that applies) | 1. Headache 2. Fever 3. Dry cough 4. Difficulty in breathing 5. Stomachache 6. Running nose 7. Fatigue 8. Dialarhoea |  |
| **Testing and willingness to be tested** | | | |
|  |  |  |  |
| 020 | Are you at risk of COVID-19? | 1. Yes 2. No 3. Don’t know |  |
| 021 | If not at risk, why? (Choose all that apply) | ………………………. |  |
| 022 | Do you know someone who has been tested positive for COVID-19? | 1. Yes 2. No |  |
| 023 | Did you come into close contact with anyone who has been tested positive for COVID-19 before his/her test? | 1. Yes 2. No 3. Don’t know |  |
| 024 | Have you been tested for COVID-19? | 1. Yes 2. No | Skip 025 |
| 025 | If not tested, are you willing to be tested for COVID-19? | 1. Yes 2. No |  |
| 026 | Will you be willing to reveal your status to friends and co-workers if tested positive for COVID-19? | 1. Yes 2. No 3. Don’t know |  |
| 027 | Will you be surprise if you are tested positive for COVID-19? | 1. Yes 2. No |  |
| 028 | Have you been ill or had an appointment to visit a health facility in the last two months? | 1. Yes 2. No | If no, skip the next two questions |
| 029 | If yes, did you visit the health facility? | 1. Yes 2. No |  |
| 030 | If no, was your decision based on the fear of contracting COVID-19 at the health facility? | 1. Yes 2. No |  |
| 031 | Do you fear to visit a health facility if you are ill because of coronavirus? | 1. Yes 2. No |  |
| **Racism, Stigma, and discrimination attitudes** | | | |
|  | Government of china should be blamed for the global spread of COVID-19 | 1. Strongly agree 2. Agree 3. Neutral 4. Strongly disagree 5. disagree |  |
|  | I will avoid sitting in the same vehicle with someone from China or Asia | 1. Strongly agree 2. Agree 3. Neutral 4. Strongly disagree   disagree |  |
|  | I will avoid sitting in the same vehicle with someone from America or Europe | 1. Strongly agree 2. Agree 3. Neutral 4. Strongly disagree   disagree |  |
|  | I will avoid sitting in the same vehicle with someone from other African country | 1. Strongly agree 2. Agree 3. Neutral 4. Strongly disagree   disagree |  |
|  | All foreigners should be made to go to their countries | 1. Strongly agree 2. Agree 3. Neutral 4. Strongly disagree   disagree |  |
|  | I will treat a COVID-19 patient if I were a Nurse or Doctor | 1. Strongly agree 2. Agree 3. Neutral 4. Strongly disagree   disagree |  |
|  | I will stay in the same house with a health worker involved in managing COVID-19 cases | 1. Strongly agree 2. Agree 3. Neutral 4. Strongly disagree   disagree |  |
|  | I will sit in the same vehicle with a health worker involved in managing COVID-19 cases | 1. Strongly agree 2. Agree 3. Neutral 4. Strongly disagree   disagree |  |
|  | I will avoid eating with a health worker involved in managing COVID-19 cases | 1. Strongly agree 2. Agree 3. Neutral 4. Strongly disagree   disagree |  |
|  | I will stay in the same house with a recovered COVID-19 patient | 1. Strongly agree 2. Agree 3. Neutral 4. Strongly disagree   disagree |  |
|  | I will be willing to sit in the same vehicle with a recovered COVID-19 patient | 1. Strongly agree 2. Agree 3. Neutral 4. Strongly disagree   disagree |  |
|  | I will avoid working together with a recovered COVID-19 patient | 1. Strongly agree 2. Agree 3. Neutral 4. Strongly disagree   disagree |  |
|  | I will avoid eating together with a recovered COVID-19 patient | 1. Strongly agree 2. Agree 3. Neutral 4. Strongly disagree   disagree |  |
|  | I would not mind caring for an infected COVID-19 patient in self isolation (e.g cooking, run errands etc.) | 1. Strongly agree 2. Agree 3. Neutral 4. Strongly disagree   disagree |  |
|  | Names of all people tested positive for COVID-19 should be made public | 1. Strongly agree 2. Agree 3. Neutral 4. Strongly disagree   disagree |  |
|  | I would not mind if the government decide to use any facility close to where I stay as isolation/quarantine centre for COVID-19 patients or suspects | 1. Strongly agree 2. Agree 3. Neutral 4. Strongly disagree   disagree |  |
|  | I will sack my worker who has been tested positive for COVID-19 and recovers from it if I am an employer | 1. Strongly agree 2. Agree 3. Neutral 4. Strongly disagree   disagree |  |
|  | I believe a recovered COVID-19 patient should not be allowed to work as (please choose all that apply) | 1. Health personnel 2. Teacher 3. waiter/waitress 4. cook 5. bus driver 6. other………………………… 7. none of the above |  |
| **Preventive Practices** | | | |
|  | What will you do if you suspect to have come into close contact with someone who has recently been test positive for COVID-19? (choose all that apply) | 1. Self-quarantine 2. call emergency line 3. visit a health facility 4. Don't know what to do 5. will do nothing 6. other…………………. |  |
|  | What will you do if you suspect to have symptoms of COVID-19? (choose all that apply) | 1. Self-quarantine 2. call emergency line 3. visit a health facility 4. Don't know what to do 5. will do nothing   other…………………. |  |
|  | Do you know the emergency number to call in case you suspect that you have symptoms of COVID-19? (please ask to mention) | 1. yes 2. No |  |
|  | In recent days, have you gone to any crowded place? | 1. Yes 2. No 3. Cannot remember |  |
|  | In recent days, have you worn a mask when leaving home? | 1. Yes 2. No   Cannot remember |  |
|  | Do you own and use hand sanitizer? | 1. Yes 2. No |  |
|  | Do you now wash your hands more frequently than before since the first case of COVID-19 was reported in Ghana? | 1. Yes 2. No 3. Don’t know |  |
